# Supplementary material for: Earliest millet cultivation reflects steppe connections, dietary flexibility, and resilience in Bronze Age northern Greece
Source: PLoS One. 2026 Mar 3;21(3):e0344099. doi: 10.1371/journal.pone.0344099 (PMC12956113; doi:10.1371/journal.pone.0344099)
Supplement: Attachment — Submitted filename: response_to_reviewers.docx [file pone.0344099.s004.docx]

**Manuscript ID:** PONE-D-25-57342

**Title:** *Earliest millet cultivation reflects steppe connections, dietary flexibility, and resilience in Bronze Age northern Greece*

**Response to the Editor and Reviewers:**

We would like to thank the Academic Editor and the reviewers for their careful reading of our manuscript and for their constructive and insightful comments. We have revised the manuscript accordingly and believe that the changes have significantly improved the clarity, structure, and robustness of the paper. Below, we respond to each comment point by point.

**Academic Editor:** We thank the Academic Editor for handling our manuscript and for the editorial guidance. In addition to addressing the reviewers’ comments, we have also revised the manuscript to fully comply with all PLOS ONE editorial requirements. All map figures have been replaced with new figures based exclusively on public-domain basemap data from Natural Earth, and the data sources are now explicitly stated in the figure captions. We have added a detailed permits and repository statement in the Methods section. Funding information has been removed from the manuscript and is now provided in full detail in the cover letter. The Data Availability Statement has been updated in accordance with PLOS ONE policy.

**Reviewer #1:**

1. The section on radiocarbon dates is extremely difficult to read in the current state. As you have perfectly layouted tables, I do not see the necessity to repeat so many of them in the text.

Thank you for this comment. We have revised the radiocarbon section by removing the sample-by-sample listing of dates from the main text and by restructuring the description to focus on the chronological patterns and their interpretation. All numerical details are now presented exclusively in Table 4, to which the text refers.

See Section: ‘’Radiocarbon dating and calibration’’ starting at line: 277 and Radiocarbon Results: starting at line 381.

2. You submitted all the tables as supporting information, and I strongly advise against this choice. The tables are small, highly informative, and in my opinion they would all perfectly fit into the document itself.

All main tables have now been moved from the Supporting Information and are included in the main manuscript.

3. Please consider merging tables 4, 5, and 6 into one table.

We agree with this suggestion. Tables 4, 5, and 6 have been combined into a single table (Table 4), so that all radiocarbon dating information is now presented in one place.

See Table 4 around line 416.

**Reviewer #2:**

1.Intro: why not include mentions on Crete as it is really part of this Mycenaean-Minoan framework?

We thank the reviewer for this comment. We agree that this was not sufficiently clear in the original version of the manuscript. We have now added a clarification in the section where the geographical terms are defined, stating that the main focus of the paper is northern Greece in relation to the southern Greek mainland, and more specifically to the Mycenaean centers there.

Crete is not included in the present discussion, as evidence for broomcorn millet on the island is extremely scarce or absent for the period in question, and no direct AMS radiocarbon dates currently exist. Moreover, given its geographical position, the question of millet in Crete would also have to be discussed, among other things, within a broader and different set of connections and exchange networks, and would require a more extensive analysis than is possible within the scope of the present paper. We are currently preparing a study with a broader geographical focus, including Anatolia, in which Crete will also be explicitly included in the discussion.

See lines: 51-54.

2. Data ARE (and not ‘is’)

Corrected throughout the manuscript.

Results -Cereals:

3. ‘Four cereal species have been identified at Skala Sotiros: millet (Panicum miliaceum), barley 255 (Hordeum vulgare), emmer (Triticum dicoccum), and einkorn (Triticum monococcum).’: from the discussion below and the table it seems that it is not clear if einkorn was present (T. mono/dico) so maybe remove it from the opening sentence or edit to reflect this. You also mention einkorn (as secure presence) in the discussion, so this needs editing too.

We have revised the text accordingly and no longer list einkorn as a securely identified crop. The cereal assemblage is now described as comprising millet, barley, and emmer, with a small number of grains recorded more cautiously as *Triticum monococcum/dicoccum*.

See lines around 288-300 and 426-428.

4. Not sure how you differentiate between H. vulgare and Hordeum sp. All barley should be H. vulgare and then you have subspecies for hexastichum or distichum. Do you mean H. vulgare as in 6-row barley? Please clarify as the different ways of nomenclature may cause confusion.

Thank you very much for pointing this out. You are right that the previous wording was unclear and misleading. The difference does not lie in taxonomic attribution, but in the state of preservation. All barley remains are attributed to *Hordeum vulgare*. Of these, 145 grains are sufficiently well preserved to be interpreted as hulled forms, while the remaining 38 grains are more poorly preserved and do not retain the necessary diagnostic morphological features for more details. The distinction between two-row and six-row barley cannot be securely made based on the assemblage.

See lines 297-300.

5. In regards to the mesh size and its potential impact: what about the wild taxa that you found? Did you find only bigger ones or did you also have small seeds? This can give you another indication on whether you lost material or not. As far as I can see most are bigger ones but maybe some of the Poaceae ones are small? Using this line of evidence you can make a comment (in all likelihood that material was lost).

Thank you for this helpful suggestion. Indeed, most of the wild taxa are represented by medium- to large-sized seeds, while Poaceae are very few. This observation confirms that smaller remains were likely underrepresented due to the recovery method. We have added this comment to the manuscript.

See lines 325-327.

6. Results overall: I find it too wordy; there is a lot of detail on the finds of each trench that is not needed. I think this information can be best summarised in a table and discuss the finds in a more concise manner in the text, which you do anyway below when you synthesise the finds, after the wild taxa. I recommend deleteing many of the details on descriptions and extend instead the discussion of the general trends of the results across period and area.

We have substantially revised and condensed the Results section by removing most of the trench-by-trench and sample-by-sample narrative description for both cereals and pulses. The main text has been rewritten in a more synthetic way to focus on overall patterns, diachronic trends, and differences between phases and trenches, while retaining the key quantitative summaries and methodological caveats. This has resulted in a shorter, clearer, and more interpretative Results section, as suggested by the reviewer.

Starting line: 287.

7. Nomenclature: you may want to use updated nomenclature. Please choose an (ideally updated) flora and add in the text which nomenclature you are following. Graminae for example are now named Poaceae, etc etc.

Yes, the nomenclature in the original version was based primarily on *Flora Europaea*; however, we have now updated and standardized the plant nomenclature following Plants of the World Online (POWO) and have added this explicitly in the Methods section of the revised manuscript.

See lines around: 252- 254 and 323.

8. Line 362: ‘burning layers’ change to ‘burnt layers’.

We have corrected this as suggested.

9. Radiocarbon results: some of this information needs to be moved to the methods section and leave here only the results.

We have moved the methodological information on radiocarbon dating to a dedicated subsection in the Study Methods section and revised the “Radiocarbon results” section so that it now presents only the results and their interpretation.

See lines 277 and 381.

10. In regards to the RC results and the discrepancy with archaeological data: it is worth considering and discussing in the text whether any of the grains (especially in the case of millet) could be later intrusions into older layers due to bioturbation or other potential taphonomic factors.

We thank the reviewer for this comment. We have now clarified this point in the Discussion.

See lines 446-449.

Discussion:

11. presence frequency (Stetigkeit): I believe you refer here to ‘ubiquity’?

Yes, the term “frequency” was used incorrectly here and has been replaced with “ubiquity” in the revised manuscript.

See line 482.

**Reviewer #3:**

Around Line 70-71:
In context with your discussion of Middle and Late Bronze Age Networks in the Aegean [and especially since you mention that “Mycenaean influence in northern Greece is only minimally attested and mainly confined to a few ceramic finds”], you may wish to discuss/reference the recent (2024) large-scale research at Adatepe, by Reinhard Jung and Hristo Popov (eds):

“Searching for Gold-Resources and Networks in the Bronze Age of the Eastern Balkans”. The complete volume is available for download: austriaca.at/0xc1aa5572 0x003f5d96.pdf

Thank you for this suggestion and for pointing us to the more recent literature, which shows that interpreting the Mycenaean presence in the north solely on the limited ceramic evidence is an oversimplified approach. We have added the reference to the recent large-scale research at Ada Tepe (Jung and Popov 2024), which provides a broader interregional framework for understanding Middle and Late Bronze Age networks and the role of northern Greece / the northern Aegean within them.

See lines: 81-85.

Around Line 91 (ff)
In context with your discussion of climate and environmental change during the Bronze Age,
and especially since you mention that “two major climatic events” (at 4.2 ka calBP and 3.2 ka calBP) are under discussion, you may like to add the proposal by Bernhard Weninger, Eelco Rohling and collegues that societally significant climate variability may also have occurred in the Aegean, and particularly in the Northern Aegean, in the transition from the Late Bronze Age to the Early Iron Age (sometimes called “Dark Ages”) at around 1050-980 BCE:
Weninger, B., Clare, L., Rohling, E.J. Bar-Yosef, O.,l Böhner, U., Budja, M., Bundschuh, M., Feurdean, A., O., Linstädter, O., Mayewski, P., Muhlenbruch, T., Reingruber, A., Rollefson, G., Schyle, D., Thissen, L., Zielhofer, C., 2009. The Impact of Rapid Climate Change on prehistoric societies during the Holocene in the Mediterranean. Documenta Praehistorica XXXVI, 7-59. DOI: 10.4312/dp.36.2
Rohling, E.J., Marino, G., Grant, K.M., Mayewski, P.A., Weninger, B., 2019.A model for archaeologically relevant Holocene climate impacts in the Aegean-Levantine region (easternmost Mediterranean). Quaternary Science Reviews, Volume 208, 2019, Pages 38-53, ISSN 0277-3791. <https://doi.org/10.1016/j.quascirev.2019.02.009>.
You might also like to add the (in my view: useful) overview of the available climate records in the Aegean (and Near Eastern) Bronze Age by:
Jacobson, M. J., Seguin, J., & Finné, M. (2024). Holocene hydroclimate synthesis of the Aegean: Diverging patterns, dry periods and implications for climate-society interactions. The Holocene, 0(0). 1-17 <https://doi.org/10.1177/09596836241275028>

We thank the reviewer for these suggested references. We have revised the climate section to incorporate recent palaeoclimatic syntheses, emphasizing regional variability and non–mono-causal interpretations of climate–society interactions, and have added appropriate references to Rohling et al. (2019) and Jacobson et al. (2024).

See lines around: 112-113 and 140-143.

Lines 390-391
You might like to abbreviate the repetitive notation of the calibrated ages by the following
method or similar:
Sample 260 (Poz-144410) produced an age of 3130 ± 35 BP: 1497–1294 cal BCE (95.4%).

We have revised the presentation of calibrated radiocarbon dates throughout the manuscript by removing repetitive listings and focusing the text on interpretation rather than numerical detail, which is in the table.

See Section: ‘’Radiocarbon results’’ starting at line: 381.
