# Supplementary material for: Earliest millet cultivation reflects steppe connections, dietary flexibility, and resilience in Bronze Age northern Greece
Source: PLoS One. 2026 Mar 3;21(3):e0344099. doi: 10.1371/journal.pone.0344099 (PMC12956113; doi:10.1371/journal.pone.0344099)
Supplement: Attachment — Submitted filename: Response_to_Reviewers_auresp_2.docx [file pone.0344099.s005.docx]

**Manuscript ID:** PONE-D-25-57342

**Title:** *Earliest millet cultivation reflects steppe connections, dietary flexibility, and resilience in Bronze Age northern Greece*

**Response to the Editor and reviewers:**

Thank you for your careful re-evaluation of our manuscript and for your helpful comment. We have revised the manuscript accordingly.

The chronological framework has been updated following the recommended references. Table 1 has been revised with respect to the Late Bronze Age, which is now presented as ending around 1200 BCE, in line with recent radiocarbon-based studies. The table caption and references were updated accordingly.

All relevant sections of the manuscript were adjusted to ensure chronological consistency. Specifically, revisions were made in the Introduction (approx. lines 30, 41, 59), in the section Regional variation: north versus south (approx. line 147), and in the Discussion (approx. lines 475–476), where the reference to Assiros Phase 5 was clarified following the suggested literature. Table 1 and its caption were revised accordingly (approx. lines 74–80).

All changes are indicated in the revised manuscript with Track Changes.

Thank you again for your consideration.

Kind regards,
Kyriaki Karanikola
(on behalf of all co-authors)
